# Putative Connections Between Nitrate Reductase *S*-Nitrosylation and NO Synthesis Under Pathogen Attacks and Abiotic Stresses

**DOI:** 10.3389/fpls.2018.00474

**Published:** 2018-04-11

**Authors:** Yu-Fan Fu, Zhong-Wei Zhang, Shu Yuan

**Affiliations:** College of Resources, Sichuan Agricultural University, Chengdu, China

**Keywords:** abiotic stress responses, nitrate reductase, NO synthesis, pathogen attacks, *S*-nitrosylation

## Abstract

Nitrate reductase (NR) is the key enzyme for nitrogen assimilation in plant cells and also works as an important enzymatic source of nitric oxide (NO), which then regulates plant growth and resistance to biotic and abiotic stresses. However, how NR activities are finely tuned to modulate these biological processes remain largely unknown. Here we present a SWISSPROT 3D analysis of different NR from plant sources indicating the possible sites of *S*-nitrosylation, and show some evidence of immunoblottings to S-nitrosated (SNO-) proteins. We also found that *S*-nitrosylation status of NR is negatively correlated with the enzyme activity. The production of NO via NR *in vitro* represents only 1% of its nitrate reduction activity, possibly due to NO generated through NR reaction may deactivate the enzyme by this *S*-nitrosylation-mediated negative-feedback regulation. NR-mediated NO generation also plays a key role in protecting plants from abiotic stresses through activating antioxidant enzymes and increasing antioxidants. Putative connections between NR *S*-nitrosylation and NO biosynthesis under pathogen attacks and abiotic stresses are discussed in this Perspective.

## Introduction

Nitrate reductase (NR) is the key enzyme for nitrogen assimilation in multiple organisms (Chamizo-Ampudia et al., 2017), which catalyzes the nitrate to nitrite reduction in plant cell cytoplasm (Kolbert et al., 2008). Nitrate, its main substrate, has been shown to be necessary for cellular-signaling and commonly distributed in multiple plant tissues (Kolbert et al., 2008).

Nitrate reductase is only active as a dimer with two subunits of approximate 100 kDa, each containing three prosthetic groups, heme *b*_557_, flavin adenine dinucleotide (FAD) and molybdenum cofactor (Campbell, 2001). The prosthetic groups are bound to separate polypeptide domains connected by flexible hinge regions. Hinge 1 is located between the heme and molybdenum domains, and hinge 2 between the FAD and heme domains (Campbell, 2001; Chamizo-Ampudia et al., 2017). Furthermore, the dimerization domain is linked to the hinge region 2 and to the molybdenum domain. They allow energetical transfer of the electron from one domain to another, starting with the electron donors NADH and NADPH toward nitrate (Campbell and Kinghorn, 1990; Chamizo-Ampudia et al., 2017).

Nitrate reductase functions as a very important enzyme for nitric oxide (NO) biosynthesis. It was established in *Chlamydomonas* that NR plays a key role in NO homeostasis by providing electrons from NADH or NADPH via its catalytic domain both to a truncated hemoglobin THB1 (Sanz-Luque et al., 2015), which functions in NO scavenging through the dioxygenase activity, and to a molybdo-enzyme NO-formation NR (NOFNiR) that is involved in its NO biosynthesis (Chamizo-Ampudia et al., 2016, 2017).

Phosphorylation modifications on NR modulate the NO generation rate (Rockel et al., 2002; Lillo et al., 2004). Besides phosphorylation, NR may be modified by the cysteine (Cys) *S*-nitrosylation on the protein surface. Here we present a SWISSPROT 3D analysis of different NR from plant sources indicating the possible sites of *S*-nitrosylation, and show an evidence of immunoblottings to S-nitrosated (SNO-) proteins to support this structure analysis. NO generated through NR may deactivate NR itself and down-regulate NO synthesis via this feedback loop.

Recent phytopathological studies suggested that pathogen-resistance-associated NO-synthesis influences nutrition homeostasis to favor nitrite diversions either away from infection sites or toward defensive-related metabolism (Mur et al., 2017). It is well known that NR participates in tissue development and abiotic stress adaption by regulating both the NO accumulation level and the metabolic flux of nitrate assimilation (Ziogas et al., 2013). *S*-nitrosylation modification on NR and its physiological significance on NO-synthesis and stress acclimation are illuminated in this Perspective.

### Possible *S*-Nitrosylation to the Cys Residues on Nitrate Reductase Protein Surface

It was well-known that for plant cells, NR is activated or inactivated by de-phosphorylation or phosphorylation modification, respectively (Lea et al., 2004). The phosphorylation site is located on a specific motif in the hinge 1 between the heme-binding domain and the molybdenum cofactor-binding domain. The phosphorylatable amino acid is Ser534 in Arabidopsis, Ser543 in spinach or Ser521 in tobacco (Bachmann et al., 1996; Su et al., 1996). Nevertheless, the post-translational regulatory mechanism is more complicated than Ser phosphorylation, because that there are also several Cysteine (Cys) residues distributed on the NR protein surface, which may be subjected to *S*-nitrosylation modification.

**FIGURE 1:**
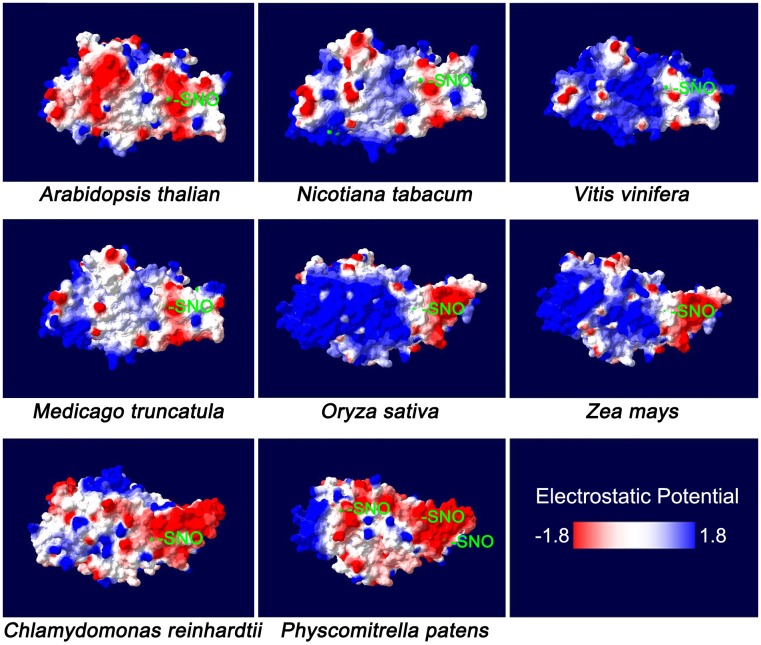
The molecular surface of plant nitrate reductase. The NR sequences of *Arabidopsis thaliana* (Accession ID: X13434.1), *Nicotiana tabacum* (XP_016462202.1), *Vitis vinifera* (XP_019082269.1), *Medicago truncatula* (XP_003614820.1), *Oryza sativa* Japonica Group (XP_015650300.1), *Zea mays* (NP_001292785.1), *Chlamydomonas reinhardtii* (AAF17595.1) and *Physcomitrella patens* (XP_001763055.1) were selected for the analysis of homology models which were constructed in the SWISS-MODEL Workspace (http://swissmodel.expasy.org/workspace/). The molecular surface and the electrostatic potential were computed with Swiss-PdbViewer v. 4.0.1 software (Biasini et al., 2014). The colors on the molecular surface indicate electrostatic potentials (red: –1.8; blue: +1.8). The cysteine (Cys) residues are marked with light-green color, some of which may be subjected to *S*-nitrosylation (-SNO).

**FIGURE 2:**
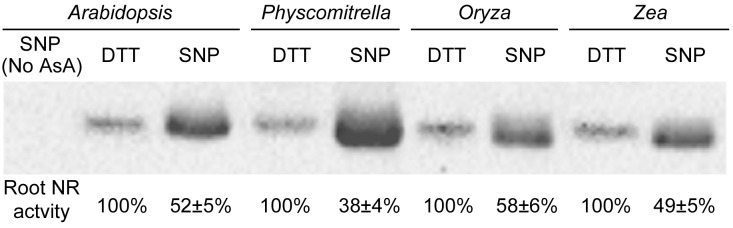
*S*-Nitrosylation levels and root enzyme activities of nitrate reductase from four plant species. The 14-day-old *Arabidopsis thaliana*, *Physcomitrella patens*, *Oryza sativa* and *Zea mays* seedlings treated grown with or without 0.2 mM SNP (sodium nitroprusside; a NO donor) or 2 mM DTT (dithiothreitol; a NO inhibitor) were harvested for protein extraction. Total cellular proteins were extracted with HEN buffer (250 mM Hepes-NaOH pH 7.7, 1 mM EDTA, 0.1 mM neocuproine and proteinase inhibitor cocktail). After placing on ice for 20 min, the homogenate was centrifuged at 4°C at 14,000 rpm for 5 min. The supernatant was placed into a new tube and centrifuged it at 4°C at 14,000 rpm for 15 min. About 200 μg total cellular proteins were diluted into 125 μl HEN buffer with 500 μl blocking buffer [2.5% SDS and 20 mM methylmethane thiosulphonate (MMTS) in HEN buffer]. After 60 min incubation in 50°C under continuous shaking, MMTS was removed by acetone precipitation. The protein was diluted with 100 μl HEN buffer plus 1% SDS. The labeling reaction was initiated by adding 25 μl of 400 μM sodium ascorbate and 25 μl biotin-M (Sigma-Aldrich) and incubating at 25°C 2 h on a shaker. All proteins after biotin-switch were immunoprecipitated with the biotin antibody (Abcam), and then were subjected to Western blotting with the Arabidopsis NR antibody (Agrisera: AS08 310). 1/10 of the immunoprecipitated proteins (corresponding to 20 μg total cellular proteins) were loaded into each lane (Zhang et al., 2017). Biotin-switch assay and Western blotting were repeated 3 times, and typical results were presented. Protein extracts without ascorbate treatments were used as the negative control (No AsA). The NR activity was measured by mixing 1 volume of protein extracts with 5 volumes of pre-warmed (25°C) assay buffer (100 mM HEPES-KOH, pH 7.5, 5 mM KNO_3_, and 0.25 mM NADH). The reaction was started by the addition of assay buffer, incubated at 25°C for 30 min, and then stopped by adding 0.1 M zinc acetate. After 15 min, the tubes were centrifuged at 13,000 ×*g* for 5 min. The nitrite produced was measured at 520 nm by adding 1 mL of 1% (w/v) sulfanilamide in 3 M HCl plus 1 mL of 0.02% (v/v) N-(1-naphthyl)-ethylenediamine (Hu et al., 2015). Root NR activity of each plant treated with DTT was normalized to 100%. Enzyme activities under the SNP treatment are shown as mean ± standard deviations (*n* = 3).

Homology models of NR protein from eight species were constructed in the SWISS-MODEL Workspace^1^. The molecular surface and the electrostatic potential were computed with Swiss-PdbViewer v.4.0.1 software (Biasini et al., 2014). Cys residues distributed on the protein surface were further analyzed (**Figure 1**). As a previous report suggested, negative or neutral electrostatic potentials on the protein surface or on the amino acid residues adjacent to the Cys residues on the protein surface are considered to be required for the *S*-nitrosylation reaction (Zhang et al., 2017). From **Figure 1**, we know that at least one Cys residue meets this requirement could be always found on the protein surface for every plant species. Therefore, *S*-nitrosylation on NR may occur presumably. The Cys *S*-nitrosylation may hamper assembly of the protein oligomer or folding of the nascent peptide chain, and therefore inhibits the enzyme activity (Lindermayr et al., 2005; Zhang et al., 2017). In other words, NO generated through NR may deactivate NR itself, which is a negative-feedback regulatory pathway and may be important for the balance between the N-mediated growth signals and the NO-mediated stress-responsive signals. We proved this hypothesis by the biotin switch assay (**Figure 2**). NO donor SNP (sodium nitroprusside) treatment significantly increased NR *S*-nitrosylation levels in both lower plants (*Physcomitrella patens*) and higher plants (*Arabidopsis thaliana*, *Oryza sativa*, and *Zea mays*). *S*-nitrosylation status of NR is positively correlated with the NO level, but negatively correlated with the enzyme activity (**Figure 2**). For example in *P. patens*, after the SNP treatment, the strongest NR *S*-nitrosylation was observed accompanying with the lowest NR activity (**Figure 2**).

The inhibitory effect of NO on NR has been reported before. Sanz-Luque et al. (2015) showed that the cytosol truncated hemoglobin, THB1, plays a key role in NO-mediated NR deactivation. THB1 has the NO di-oxygenase activity and gets the electron from the FAD group on NR (the di-aphorase activity), which then reduces the heme group. In the presence of NO, the reduced THB1 may catalyze NO conversion into NO_3_^-^ and inhibit NO_3_^-^ reduction by decreasing the reducing power available to the enzyme (Sanz-Luque et al., 2015). Similar *S*-nitrosylation-mediated negative-feedback regulations to nitrate transporters and reductases involved in nitrate assimilation and NO production have been proposed before (Frungillo et al., 2016). Putative correlations between THB1-mediated NR deactivation and *S*-nitrosylation-mediated negative-feedback regulatory pathway to NR need further investigations.

It is well-known that the plants preferring nitrate show higher NR activities; in contrast, ammonium-adsorbing plants present lower NR activities, particularly in roots (Marwaha and Juliano, 1976). For instance, although rice (in favor of NH_4_^+^) nitrite reductase activities are higher in the seedlings grown in nitrate than in those grown in ammonium, its root NR activity is considerably low (Marwaha and Juliano, 1976). So its ability of using NO_3_^-^ may be lower than the plants in favor of NO_3_^-^, which may be connected with different densities of NR distributed in the roots of different species (Marwaha and Juliano, 1976).

### The Relationship Between Nitrate Reductase and Nitric Oxide Biosynthesis

Nitric oxide synthesis in plants is a highly controversial research field (Corpas and Barroso, 2017; Santolini et al., 2017). Nitric oxide synthase (NOS) activity could be detected in fungi and some higher plants. Despite evidences of the arginine-dependent pathway for NO synthesis in higher plants, no NOS homolog has been identified in plants (Corpas and Barroso, 2017; Santolini et al., 2017). *Nitric Oxide Associated 1* (*NOA1*) was originally reported to encode a gene of NOS (Guo et al., 2003). However, the accumulated evidence shows that NOA1 has a distinct function from the direct NO synthesis (Corpas and Barroso, 2017; Santolini et al., 2017). Whereas there are evidences for enzymatic synthesis of NO by NR, it has been suggested that NR’s main role is to provide nitrite, which in turn can be further reduced to NO in other cellular compartments (Rockel et al., 2002). The facts that NO could be generated through nitrite by NR and also by the orthologous enzymes from bacteria, fungi and green algae (Yamasaki et al., 1999; Marcos et al., 2016; Chamizo-Ampudia et al., 2017), thus indicating that this reaction may be conserved and more general than suspected. Although data supporting the relation between NO and NR production in plant cells have steadily accumulated (Hao et al., 2010; Mur et al., 2013; Lu et al., 2014; Yuan et al., 2016), important issues remain unresolved, such as the low rate of NO production by NR and the generally unclear correlation between NR activity and NO producing efficiency. In fact, the production of NO via NR *in vitro* represents only 1% of its nitrate reduction activity at saturating nitrite and NADH contents, and it could be inhibited by the nitrate competitively (Gupta et al., 2011). Not with standing, the cytosol nitrate level is about the mmol range, so the NO generation from nitrite would be very inefficient, unless NR was exponentially activated (Miller and Smith, 2008). One of the reasons may be that NO generated through NR deactivates NR itself by the *S*-nitrosylation feedback inhibitions of nitrate transporters and reductases. Therefore, we should not overlook the NOS-like pathways. Although it is a yet cryptic pathway, synthesis of NO from L-arginine intuitively goes side-by-side with the nitrate assimilatory pathway.

### Nitrate Reductase Is Involved in Pathogen Resistance

Nitrate assimilatory pathway involves a lot of reductases after the *trans*-amination process, which is responsible for the amino acid biosynthesis (Lam et al., 1996). When pathogens come into (infect) the plant tissues, they are often nutritional-starved such that fast nitrate assimilation from the hosts is necessary for the valid infection (López-Berges et al., 2010). On the other hand, plants may re-allocate their nutrients to the defensive responses or away from the infection sites (Mur et al., 2017). Exogenous applications of N fertilizer could thus change the balance between the hosts and the pathogens. The increases of N have been suggested either to enhance or to reduce plant’s resistances to biotic stresses (Robert et al., 2002; Solomon et al., 2003), reflecting the difference in defensive strategies of different plants (Mur et al., 2017). Nitrate or ammonium fertilizers affect the outcomes of pathogen-plant interactions differently. In general, nitrate feeding enhances pathogen resistance, while ammonium compromises the defensive response (Mur et al., 2017). Metabolically, NO_3_^-^ enhances polyamine production, which is a well-known defense signal. While NH_4_^+^ nutrition leads to an increase in γ-aminobutyric acid, which might be a nutritional component used by some pathogens (Abrahamian et al., 2016).

Companying with the defensive N metabolism, the role of NO should also be considered. It has been suggested that both nitrate and ammonium could increase NR activity and the related NO production (Gupta et al., 2005; Planchet et al., 2005). The pathogen-induced NO accumulation influences the N homeostasis to favor nitrite diversions either away from infection sites or toward defensive-related metabolism (Mur et al., 2017). This NO accumulation is mainly derived from NR and is promoted by both Pathogen Associated Molecular Pattern (PAMP) and gene-to-gene mediated defense. NO accumulation and the correlated defense responses are nitrate dependent and are therefore largely compromised by ammonium (Mur et al., 2017). Yamamoto-Katou et al. (2006) indicated that silence of NR resulted in decreased NO contents after the elicitin treatment; these proteins are secreted by *Phytophthora* and *Pythium* species during the phytopathogenic attack. A recent report indicated that, during the compatible interaction between tobacco and *Pseudomonas syringae*, the mitochondrial NO production via NR-mediated mechanism increased plant resistance to pathogens (Gupta et al., 2013). Detailed biochemical study revealed that the *P. syringae* infection induced mitochondrial NO accumulation and nitrosylation of the photo-respiratory glycine decarboxylase complex (GDC) in mitochondria (Palmieri et al., 2010). Considering that NADH is needed for the redox balance, nitrosylation results in GDC inhibition, which could influence overall redox homeostasis and induce the cell-death (Palmieri et al., 2010).

### Nitrate Reductase Participates in Abiotic Stress Adaptation

Despite these negative arguments for the role of NR in NO production, many studies have been confirmed that NR is involved in plant development and stress responses by modulating not only the content of the signaling molecule NO but also the level of nitrite available for nitrate assimilation (Campbell, 2001). NO not only leads to the production of peroxy-nitrite and the subsequent cellular damages, but also functions as a molecule in signaling, regulating stress responses and controlling stomatal closure (Garcia-Mata and Lamattina, 2003). Recent study demonstrated that NR affects potassium nutrition status as well as NO-mediated regulation to guard cell ion channels in Arabidopsis (Chen et al., 2016). NR also drives re-partitioning of carbon flux by regulating genes encoding enzymes for chlorophyll biosynthesis and carbon fixation and metabolism in the model pennate diatom *Phaeodactylum tricornutum* (McCarthy et al., 2017). Therefore NR plays a key role in controlling plant nutritional stress acclimations.

Nitrate reductase is correlated with NO synthesis and NO is a stress signaling molecule. Therefore they regulate plant acclimation to adverse environments (Chamizo-Ampudia et al., 2017). For example, a recent study indicated that NR-mediated early NO generation in the shoots of hulless barley plays an irreplaceable role in protecting seedlings from copper toxicity through enhancing antioxidant enzyme activities and antioxidant levels (Hu et al., 2015). It is interesting that the early NO burst at 24 h was produced through NR, but not through the NO synthase (Hu et al., 2015).

It was also recognized that auxin shares common steps with NO in the signal transduction cascade toward the auxin-induced adventitious and lateral root formation (Lamattina et al., 2003; Correa-Aragunde et al., 2004). This increased level of NO was present only in the lateral-root initials in contrast to the primary roots where it remained at the control level. Thus, NR is involved in auxin-induced NO formation in roots (Kolbert et al., 2008) and participates in biotic and abiotic stress responses indirectly.

An interesting study showed that in a NR-null mutant, the auxin receptor *AFB3* mRNA could be induced by the NO_3_^-^ treatment with the maximum level at 1 h, as well as the wild-type seedlings. However, the *AFB3* transcript did not decrease after that time in the Arabidopsis *nr* mutant under the same conditions, contrasting to the wild-type seedlings (Vidal et al., 2010). Therefore, besides auxin signaling, NR may also regulate auxin metabolism. The authors suggested that some N metabolite downstream of nitrate reduction may be involved in *AFB3* gene expression regulation (Vidal et al., 2010), which requires further explorations. These results could be ascribed to the role of NR-mediated NO production. Crosstalk between NO signaling/metabolism and auxin signaling/metabolism in nutritional stress responses also needs further investigations.

## Concluding Remarks

Nitrate reductase is an important enzymatic source of biosynthesis of NO. While NO functions as a signaling molecule, regulating plant development and growth, resistance to abiotic and biotic stresses and metabolic repartition for optimizing nutrient utilization. Analysis to Cys residues distributed on the protein surface implies the possible *S*-nitrosylation (by the free radical NO) on NR protein. Understanding how NR activities are finely tuned to modulate these biological processes would require future researches.

Although NO generated through NR may deactivate NR itself by the *S*-nitrosylation feedback inhibition, NR-mediated NO production is a common plant’s resistance strategy against multiple biotic and abiotic stresses (Baudouin and Hancock, 2014; Simontacchi et al., 2015; Zhang et al., 2017). Besides these signaling events and developmental processes, NO induces the post-translational modification of target proteins by NO-dependent protein nitrosylation. However, a study from Ziogas et al. (2013) showed that in citrus plants, the *S*-nitrosylation pattern was enhanced by drought, cold or heat, but was suppressed by dark or salinity. Correspondingly, peroxynitrite (ONOO^-^) scavenging ability of these plants was elicited by dark, continuous-light or drought but was suppressed by the salt stress. In contrast, nitration levels were elevated by salinity and suppressed by dark or continuous-light. They suggested that the nitrosative responses of citrus plants may be differentially regulated depending on how they are stressed. Alternatively, the roles of NR-mediated NO production and its related protein nitrosylation on biotic and abiotic stress resistance may be varied depending on the stress type or the plant species, which needs further studies.

## Author Contributions

Y-FF and SY conceived the perspective, collected data, and wrote the manuscript. Z-WZ helped to write the manuscript.

## Conflict of Interest Statement

The authors declare that the research was conducted in the absence of any commercial or financial relationships that could be construed as a potential conflict of interest.

## Footnotes

1 http://swissmodel.expasy.org/workspace/
